# Protective Effects of a Novel *Lactobacillus brevis* Strain with Probiotic Characteristics against *Staphylococcus aureus* Lipoteichoic Acid-Induced Intestinal Inflammatory Response

**DOI:** 10.4014/jmb.2110.10034

**Published:** 2021-11-06

**Authors:** Won-Ju Kim, Jun-Hyun Hyun, Na-Kyoung Lee, Hyun-Dong Paik

**Affiliations:** Department of Food Science and Biotechnology of Animal Resources, Konkuk University, Seoul 05029, Republic of Korea

**Keywords:** Probiotics, anti-inflammatory, HT-29 cell, ERK signaling, Akt signaling

## Abstract

Probiotics can effectively modulate host immune responses and prevent gastrointestinal diseases. The objective of this study was to investigate the probiotic characteristics of *Lactobacillus brevis* KU15152 isolated from kimchi and its protective potential against intestinal inflammation induced by *Staphylococcus aureus* lipoteichoic acid (aLTA). *L. brevis* KU15152 exhibited a high survival rate in artificial gastric and bile environments. Additionally, the adhesion capability of the strain to HT-29 cells was higher than that of *L. rhamnosus* GG. *L. brevis* KU15152 did not produce harmful enzymes, such as &beta;-glucuronidase, indicating that it could be used as a potential probiotic. The anti-inflammatory potential of *L. brevis* KU15152 was determined in HT-29 cells. Treatment with *L. brevis* KU15152 suppressed the production of interleukin-8 without inducing significant cytotoxicity. The downregulatory effects of *L. brevis* KU15152 were involved in the suppression of nuclear factor-kappa B activation mediated by the extracellular signal-regulated kinase and Akt signaling pathways. Collectively, these data suggest that *L. brevis* KU15152 can be used in developing therapeutic and prophylactic products to manage and treat aLTA-induced intestinal damage.

## Introduction

The human gut microbiota comprises trillions of microorganisms. This microbiota is essential for nutrient absorption, gut barrier function, and immunomodulation [1]. Lipopolysaccharide (LPS) or proinflammatory cytokines stimulate intestinal epithelial cells, resulting in intestinal inflammation. Gut microbiota act as barriers against external stimulation bacteria. Aberrant microbiota can allow the invasion of pathogenic bacteria, leading to the activation of a damaging immune response and various intestinal disorders [2]. Lipoteichoic acid (LTA) is a cell wall component of gram-positive bacteria. LTA is considered comparable to LPS, because of its physiological and biochemical properties. Inflammatory responses can be triggered by either LPS or LTA, resulting in tissue damage and organ failure [3]. However, immunomodulatory effects of LTAs from different strains can be distinct [4].

Probiotics are live microorganisms that are beneficial to host health when ingested in adequate amounts. As natural inhabitants of gut, probiotics can modulate the microflora and protect the gut against invading pathogens [5]. Probiotics maintain mucosal or systemic immune responses and promote barrier function [6]. In addition, probiotics can downregulate the expression of proinflammatory cytokines and ameliorate intestinal inflammatory diseases by suppressing nuclear factor-kappa B (NF-κB) signaling pathway [7, 8].

Recent studies have focused on the functions of lactic acid bacteria (LAB), including *Lactobacillus* sp., *Lactococcus* sp., *Weissella* sp., and *Leuconostoc* sp., which are the dominant strains in the microflora of kimchi, a traditional Korean fermented food [9, 10]. Kimchi is recognized as contributing to the health of individuals. During fermentation, LAB strains produce the unique flavor of kimchi and lactic acid, which decreases the pH of the product [11]. Several LAB strains have probiotic characteristics and various beneficial properties such as antioxidant, antimicrobial, and immunostimulatory activities [12]. In particular, they participate in the maintenance and modulation of homeostasis in the intestinal immune system [13]. *Lactobacillus* species regulate inflammatory responses by inhibiting interleukin (IL)-8 production and NF-κB activation in intestinal epithelial cells [14]. The present study aimed to investigate the probiotic potential of a novel *Lactobacillus* strain, *L. brevis* KU15152, and the effects of the strain in alleviating *Staphylococcus aureus* lipoteichoic acid (aLTA)-induced intestinal inflammatory responses.

## Materials and Methods

### Chemicals and Reagents

De Man, Rogosa, and Sharpe (MRS) medium was purchased from Difco Laboratories (USA). Roswell Park Memorial Institute (RPMI) 1640 medium, antibiotics solution, and phosphate-buffered saline (PBS) were obtained from HyClone Laboratories, Inc. (USA). Fetal bovine serum (FBS) was purchased from Life Technologies (USA). Equipment and reagents for western blotting were purchased from Bio-Rad (USA). Other chemicals including aLTA were purchased from Sigma-Aldrich (USA).

### Bacterial Strains and Cell Culture Conditions

*L. brevis* KU15152 was isolated from Chonggak kimchi. *L. rhamnosus* GG (LGG), used for comparative analysis, was obtained from the Korean Collection for Type Cultures (Korea). *L. brevis* KU15153 was used as a comparative strain owing to its anti-inflammatory effects [15]. To evaluate the protective effect of these strains, *L. brevis* strains and LGG were incubated in MRS at 37°C for 18 h. The bacteria were recovered by centrifugation (12,000 ×*g*, 4°C, 5 min), washed twice with PBS, and suspended in RPMI 1640 medium at 7 log colony forming units (CFU)/ml. These are defined as the bacterial sample.

HT-29 cells obtained from the Korean Cell Line Bank (Korea) were cultured in RPMI 1640 medium supplemented with 10% FBS and 1% penicillin-streptomycin solution. Cells were maintained at 37°C in a 5%CO_2_-containing humidified atmosphere and sub-cultured at 70–80% confluency.

### Tolerance to Artificial Gastric Juice and Bile Salts

The tolerance of *Lactobacillus* strains to gastrointestinal conditions was determined as previously described [11]. Briefly, *L. brevis* KU15152 and LGG were pre-cultured overnight in MRS medium. To determine their resistance to artificial gastric juice and bile salts, these strains were incubated in MRS broth containing 0.3% (w/v) pepsin (pH 2.5) for 3 h, and MRS broth with 0.3% (w/v) oxgall for 24 h, respectively. The survival rate (%) of the strains was calculated as follows:

$$\text{Survival rate(\%) = }\frac{\text{Cell number after incubation (log CFU/ml)}}{\text{Inoculated cell number (log CFU/ml)}}\times 100$$

### Adhesion Ability

Adhesion ability of the *Lactobacillus* strains was evaluated according to a previously described method [16] using HT-29 cells with some modifications. The cells (2 × 10^5^ per/well) were plated in 24-well plates and incubated until a confluent monolayer was obtained. Then, the medium was replaced by antibiotic-free RPMI 1640 medium. *L. brevis* KU15152 or LGG (7 log CFU/ml) were added to each well and incubated for 2 h. Non-adherent bacteria were removed by washing the wells twice with PBS. The remaining attached bacteria were collected by treatment with 0.1% Triton X-100. The adhesion ability (%) of the strains was calculated as follows:

$$\text{Adhesion ability (\%) = }\frac{\text{Attached bacterial cell number (log CFU/ml)}}{\text{Inoculated bacterial cell number (log CFU/ml)}}\times 100$$

### Enzyme Production

The ZYM kit (API, France) was used to analyze the enzyme production of the *Lactobacillus* strains, according to the manufacturer’s instructions. Overnight incubated *L. brevis* KU15152 and LGG were diluted in PBS at 9 log CFU/ml, and then added to cupules. Following incubation for 4 h at 37°C, the ZYM test reagents were added. Enzyme production was determined as the color change.

### Determination of Cell Viability

The viability of HT-29 cells was determined using the 3-(4,5-dimethylthiazol-2-yl)-2,5-diphenyltetrazolium bromide (MTT) assay with modifications [17]. Briefly, HT-29 cells (2 × 10^5^ cells/well) were seeded in 24-well plates and incubated until monolayer formation. The cells were cultured with the bacterial samples for 2 h with or without 50 μg/ml aLTA for another 22 h. MTT solution was added to each well at a final concentration of 0.5 mg/ml. The cells were incubated for 30 min, the supernatant was discarded, and the resulting formazan deposits were dissolved in 1 ml dimethyl sulfoxide. The absorbance of the resultant solution was estimated at 570 nm using Thermo Scientific Multiskan GO (Thermo Scientific, USA). Cell viability was determined as a percentage relative to the absorbance of the control groups.

### Measurement of IL-8 Production

The effect of *Lactobacillus* strains on the production of IL-8 was investigated in aLTA-induced HT-29 cells using an enzyme-linked immunosorbent assay kit (Thermo Fisher Scientific). HT-29 cells (2 × 10^5^ cells/well) were plated in 24 well plates and grown until confluent monolayers were formed. The cells were pretreated with the bacterial samples. After incubation for 2 h, cells were incubated with or without 50 μg/ml aLTA for an additional 22 h. Collected cell culture supernatants were diluted appropriately with cell growth media and used to measure the amount of IL-8 according to the manufacturer’s instructions.

### Western Blotting

Protein expression was determined using western blotting [18]. HT-29 cells (5 × 10^5^ cells/well) were plated in 6-well plates and incubated until a monolayer was formed. After 2 h of pretreatment with the bacterial samples, the cells were left untreated or stimulated with aLTA (50 μg/ml) for the indicated time. The cells were rinsed with ice-cold PBS and lysed with Pro-Prep lysis buffer (iNtRON Biotechnology, Korea) supplemented with protease and phosphatase inhibitor cocktails. The supernatant was collected from the lysates by centrifugation (15,000 ×*g*, 30 min, 4°C). Cellular proteins (20–30 μg) quantified using the DC Protein Assay Kit (Bio-Rad) were separated via 10% sodium dodecyl sulfate-polyacrylamide gel electrophoresis and transferred to a polyvinyl difluoride membrane. The membranes were blocked with 5% skim milk for 1 h and incubated with appropriate primary antibodies at 4°C overnight. Primary antibodies against phosphorylated mitogen-activated protein kinase kinase (p-MEK), extracellular signal-regulated kinase (ERK), p-ERK, Akt, p-Akt, glycogen synthase kinase (GSK) 3β, p-GSK 3β and p65 were purchased from Cell Signaling Technology, Inc. (USA). Other primary antibodies were purchased from Santa Cruz Biotechnology (USA). The membranes were then probed with horseradish peroxidase-conjugated secondary antibodies for 2 h, at room temperature. The enhanced chemiluminescence detection kit (Bio-Rad) was used to detect the protein bands, and the density of the protein bands was quantified using the ImageJ software (National Institutes of Health, USA).

### Statistical Analysis

Experimental data are presented as mean ± standard deviation (three replicates). IBM SPSS (Version 18.0, SPSS Inc., USA) was used for the statistical analysis. Statistical differences between two groups were analyzed using the unpaired one-tailed Student’s *t*-test, and those among multiple groups, using one-way analysis of variance and Tukey’s HSD test (*p* < 0.05).

## Results

### Tolerance to Artificial Digestive Conditions and Adhesion to HT-29 Cells

Resistance to artificial gastric juice and bile salt is necessary for LAB to function as probiotics. The tolerance to artificial digestive conditions is shown in Table 1. The survival rates of LGG and *L. brevis* KU15152 after exposure to artificial gastric acid (pH 2.5, 0.3% pepsin) for 3 h were 98.35% and 99.36%, respectively. In addition, *L. brevis* KU15152 exhibited high tolerance to artificial bile salt conditions (0.3% oxgall) with a viability rate of 108.24%. The rate was higher than that of LGG (100.48%).

Adhesion to intestinal epithelial cells is an important criterion for identifying potential probiotic bacteria. The adhesion of LGG and *L. brevis* KU15152 to HT-29 intestinal cells was investigated (Table 1). The adhesion rates of the two strains were 12.04% and 12.92%, respectively. The results implicated *L. brevis* KU15152 as a potential probiotic.

### Enzyme Production

The enzyme production of the LAB strains was assessed using the API ZYM kit (Table 2). Compared to LGG, *L. brevis* KU15152 did not produce α-galactosidase and N-acetyl-β-glucosaminidase. However, *L. brevis* KU15152 produced a remarkably higher amount of α-glucosidase, which catalytically mediates the bioconversion process. Additionally, the strain did not produce β-glucuronidase which is considered as a carcinogenic enzyme.

### Effect of *L. brevis* KU15152 on IL-8 Production in Intestinal Cells

**Fig. 1:**
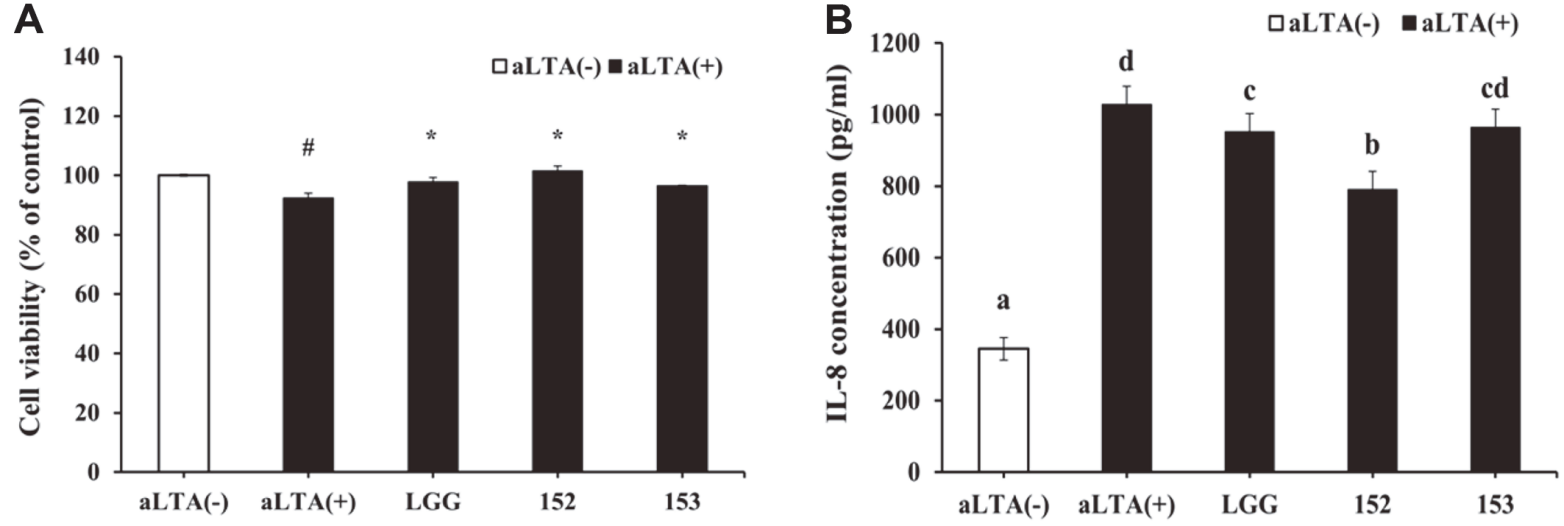
Inhibitory effect of *Lactobacillus* strains on the production of IL-8 in HT-29 cells. Cells were incubated to achieve confluence before being stimulated with aLTA (50 μg/ml) in the presence of the bacterial samples for 24 h. **A**. Viability of HT-29 cells examined using the MTT assay. **B**. IL-8 production in aLTA-induced HT-29 cells. #*p* < 0.001 compared with aLTA-negative group, *p < 0.001 compared with aLTA-positive group. Different letters indicate statistical differences (*p* < 0.05).

To investigate the effect of LTA from *S. aureus* (aLTA) and *Lactobacillus* strains with probiotic characteristics on inflammatory responses in intestinal cells, HT-29 cells were stimulated with aLTA in the presence of *Lactobacillus* strains. When treated with 8 log CFU/ml *L. brevis* KU15152, viability was 86.97 ± 1.60%. However, none of the tested groups (7 log CFU/ml) showed significant cytotoxicity in HT-29 cells (Fig. 1A). Compared to the aLTA-negative group, treatment with 50 μg/ml aLTA markedly increased the production of IL-8. However, pretreatment with *Lactobacillus* strains suppressed the production of IL-8 (Fig. 1B). Additionally, *L. brevis* KU15152 exhibited the most significant downregulation of IL-8 secretion. These findings suggest that *L. brevis* KU15152 inhibits IL-8 production, compared to *L. brevis* KU15153 and LGG without adversely affecting cell viability.

### Effect of *L. brevis* KU15152 on NF-κB Activation in Intestinal Epithelial Cells

**Fig. 2:**
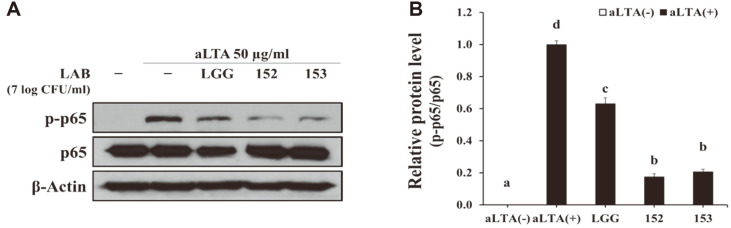
Effect of *Lactobacillus* strains on the activation of NF-κB in HT-29 cells. Cells were cultured to form a monolayer prior to treatment with *Lactobacillus* strains for 2 h. The cells were stimulated with 50 μg/ml of aLTA for another 12 h. **A**. Protein expression levels of NF-κB determined using western blotting. **B**. Relative protein expression levels of phosphorylated p65. Different letters indicate statistical differences (*p* < 0.05).

*L. brevis* KU15152 suppressed the production of IL-8 in aLTA-stimulated HT-29 cells (Fig. 1). Thus, the effect of *Lactobacillus* strains on the activation of NF-κB was determined. Cells incubated with 50 μg/ml aLTA showed markedly increased phosphorylation of p65 compared to cells incubated in the absence of aLTA. In contrast, the phosphorylation of p65 was downregulated by pretreatment with the *Lactobacillus* strains (Fig. 2). In particular, *L. brevis* KU15152 significantly inhibited the activation of NF-κB in HT-29 cells, indicating that the inhibitory effect of *L. brevis* KU15152 on aLTA-induced inflammatory response was associated with the downregulation of NF-κB activation.

### Effect of *L. brevis* KU15152 on MEK/ERK Signaling Pathways in HT-29 Cells

**Fig. 3:**
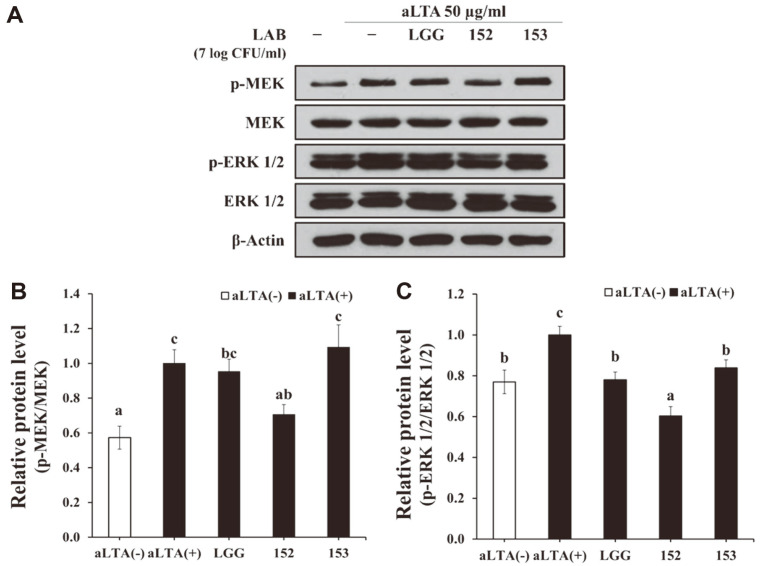
Modulatory effect of *Lactobacillus* strains on MEK/ERK signaling pathways in HT-29 cells. Fully confluent cells were pretreated with 7 log CFU/ml of *Lactobacillus* strains for 2 h and stimulated with 50 μg/ml aLTA for 20 min. **A**. Expression of phosphorylation of MEK and ERK 1/2 assessed using western blotting. **B, C.** Relative expression levels of phosphorylated MEK and ERK 1/2 quantified using the ImageJ software. Different letters indicate statistical differences (*p* < 0.05).

To determine the signaling mechanisms related to the effect of *L. brevis* KU15152 on the inflammatory response in intestinal epithelial cells, the expression levels of the MEK/ERK signaling-related proteins were examined (Fig. 3). Stimulation with aLTA significantly upregulated the expression of phosphorylated MEK and ERK 1/2 in HT-29 cells. However, MEK and ERK 1/2 phosphorylation was alleviated by treatment with *L. brevis* KU15152 for 20 min.

### Effect of *L. brevis* KU15152 on Akt/GSK 3β Signaling Pathways in HT-29 Cells

**Fig. 4:**
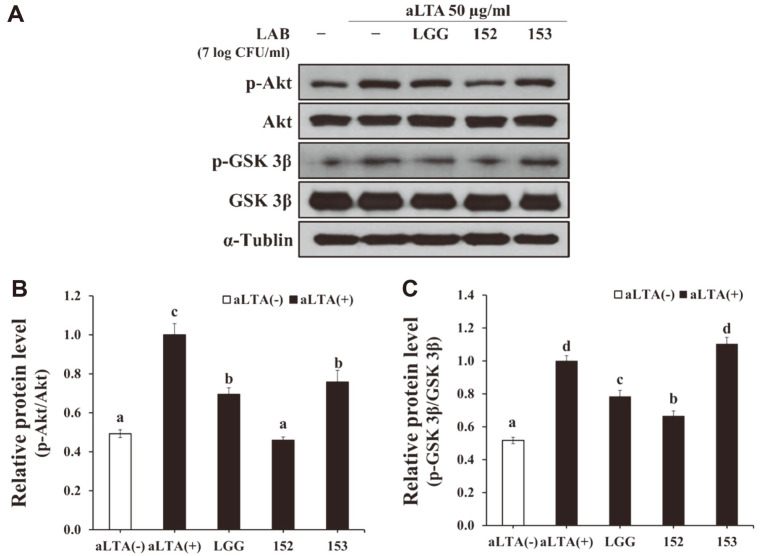
Regulatory effect of *Lactobacillus* strains on Akt/GSK 3β signaling pathways in HT-29 cells. HT-29 cell monolayers were pretreated with various *Lactobacillus* strains, followed by aLTA stimulation for 10 min. **A**. Effect of *Lactobacillus* strains on the activation of Akt/GSK 3β signaling pathways. **B, C.** Relative expression levels of phosphorylated Akt and GSK 3β. Different letters indicate statistical differences (*p* < 0.05).

IL-8 is reportedly associated with the activation of Akt, which leads to phosphorylation of GSK 3β. As shown in Fig. 4, aLTA stimulation promoted the phosphorylation of Akt. Treatment with *L. brevis* KU15152 reduced the phosphorylation of Akt induced by aLTA. The aLTA-induced activation of GSK 3β was mitigated by *L. brevis* KU15152. These findings indicate that *L. brevis* KU15152 can suppress inflammatory responses in intestinal epithelial cells by regulating the Akt/GSK 3β signaling pathways.

## Discussion

Probiotics are used to maintain and improve human health. To function as probiotics, LAB strains should survive the transit through the gastrointestinal tract and adhere to the intestine for a certain period [19]. In the present study, we demonstrated that *L. brevis* KU15152 had a high survival rate of 99.36% after incubation at pH 2.5 for 3 h. The tolerance to the gastrointestinal conditions of low pH (pH 2.5–3.5) is essential for probiotic strains, because they must go through the stomach [20]. The pH of gastric acid is usually 2.5, which can disrupt the bacterial cell envelope. Bile has a bacteriostatic role within the gut, and its effects are regulated by bile salts. However, bile salts can damage the structure of the bacterial cell membrane and kill bacteria [21]. The present study findings demonstrated the capability of *L. brevis* KU15152 to survive under condition mimicking bile salt with essentially no decline in viability after incubation in MRS broth with 0.3% oxgall for 24 h. These results are comparable with those of a previous study on the probiotic characteristics of *L. brevis* strains [15]. Furthermore, bile salt hydrolase produced by *Lactobacillus* bacteria increases the number of viable bacteria after incubation with bile salts [22]. As previously reported, probiotic organisms compete with pathogens for binding sites in the intestine, and for probiotics, intestinal adhesion is an important criterion [23]. *L. brevis* KU15152 and LGG exhibited adhesion rates of 12.92% and 12.04%, respectively. Compared with the adhesion abilities of various probiotic strains in a previous study [24], *L. brevis* KU15152 avidly adhered to intestinal epithelial cells. The strain produced various enzymes but did not produce hazardous enzymes including β-glucuronidase. These results indicate the likelihood that *L. brevis* KU15152 can survive in the human gastrointestinal tract and function as a probiotic.

LTA from *S. aureus* stimulates host cells to produce inflammatory cytokines such as IL-8 and tumor necrosis factor-α by the activation of NF-κB [25, 26]. LTAs from pathogenic bacteria, including *S. aureus*, *S. epidermidis*, and *Streptococcus pneumonia* could activate macrophages. In contrast, LTAs from probiotics and non-pathogenic bacteria, such as *L. plantarum*, have weak immunostimulatory effects [27]. In the present study, 50 μg/ml aLTA stimulated the secretion of IL-8 from HT-29 cells following NF-κB activation, and *L. brevis* KU15152 had an inhibitory effect on this activation. IL-8 is a chemokine that attracts leukocytes, such as basophils and neutrophils, to lesions and is associated with angiogenesis, metastasis, and inflammation [28]. After proinflammatory stimulation, a signaling cascade begins, leading to the translocation of NF-κB into the nucleus. In a previous study, decreased IL-8 production after glutamine pretreatment in human intestinal epithelial cells was accompanied by a reduction in the ubiquitination of IκB-α, cellular inhibitor of NF-κB [29].

In epithelial cells, *Chlamydia trachomatis* infection upregulates IL-8 secretion through the ERK signaling pathway [30, 31]. MEK1 and MEK2 are associated with the regulation of cellular processes [32]. MEK plays a role in the initiation of the MEK/ERK pathway. In addition, ERK 1/2 reacts with various substrates in the cytoplasm and nucleus and activated ERK catalyzes the activation of transcription factors, which results in the expression of several genes related to cell differentiation, proliferation, and apoptosis [33]. In the present study, the expression levels of phosphorylated MEK and ERK 1/2 increased in aLTA-stimulated HT-29 cells compared to those in the aLTA-negative group. *L. brevis* KU15152 pretreatment downregulated such expression.

The binding of IL-8 to its receptors activates the Akt signaling pathway [34]. Akt can activate B-cell lymphoma-related X protein and decrease mitochondrial membrane translocation and apoptosis [35]. Akt phosphorylates GSK 3β, which is important in cell death. For example, increased phosphorylated GSK 3β can cause apoptosis in neuronal PC12 cells and fibroblasts [36]. Furthermore, ginsenoside Rg1 modulates innate immune responses by regulating the PI3K/Akt pathway [37]. In the present study, pretreatment with *L. brevis* KU15152 reduced the extent of phosphorylation of Akt and GSK 3β induced by aLTA in intestinal epithelial cells.

In conclusion, this study demonstrates the anti-inflammatory potential and probiotic characteristics of *L. brevis* KU15152 using the representative intestinal epithelial cells, HT-29. *L. brevis* KU15152 tolerated artificial gastric acid and bile salts, and avidly adhered to HT-29 cells. *L. brevis* KU15152 alleviated aLTA-induced IL-8 production by suppressing NF-κB activation. The inhibitory effect of *L. brevis* KU15152 on intestinal inflammatory response was mediated by the regulation of MEK/ERK and Akt/GSK 3β signaling pathways. Although further studies are required, these findings provide underlying molecular evidence for the protective effects of probiotics on the intestine. The collective findings indicate the potential of *L. brevis* KU15152 as a functional probiotic product.

## Figures and Tables

**Table 1:** Tolerance of LGG and *L. brevis* KU15152 to artificial gastric acid and bile salts, and their adhesion to HT-29 cells.

Treatment	Cell number (log CFU/ml)	

	LGG	*L. brevis* KU15152
Tolerance to artificial gastric acid and bile salt		
Initial cell number	8.37±0.14	7.68±0.15
Cell number after incubation (0.3% pepsin, pH 2.5, 3 h)	8.23±0.11	7.63±0.09
Survival rate (%)	98.35±2.65^a^	99.36±0.81^a^
Cell number after incubation (0.3% oxgall, 24 h)	8.41±0.17	8.31±0.15
Survival rate (%)	100.48±1.11^a^	108.24±3.74^b^
Adhesion to HT-29 cells		
Initial cell number	8.37±0.01	7.72±0.03
Adherent cell number after 2 h	7.44±0.05	6.82±0.12
Adhesion ability (%)	12.04±1.71^a^	12.92±2.61^a^

^a,b^Different letters in the same row indicate statistical significance based on Tukey’s HSD test (*p* < 0.05).

**Table 2:** Enzyme production by LGG and *L. brevis* KU15152.

Enzymes	Enzyme Activity	

	LGG	*L. brevis* KU15152
Control	−	−
Esterase (C4)	+	+
Esterase lipase (C8)	+	+
Leucine arylamidase	+	+
Valine arylamidase	+	+
Cystine arylamidase	+	+
Acid phosphatase	+	+
Naphthol-AS-BI-phosphohydrolase	+	+
α-Galactosidase	+	−
β-Galactosidase	+	+
β-Glucuronidase	−	−
α-Glucosidase	−	+
β-Glucosidase	+	+
N-Acetyl-β-glucosaminidase	+	−

+, Production; −, Non-production.
